# Catching Disease

**Published:** 1924-03

**Authors:** Robert Blackie

**Affiliations:** National Liberal Club, S.W. 1


### "Catching Disease."

Sir,—I note your Note in the February issue, "Is Appendicitis Catching"? If there is anything in suggestion I can easily imagine it spreading by contact with those who have suffered and are afraid of it. For example, I met a doctor who was afraid to eat anything containing pips or seeds or swallowing a hair out of his tooth brush. The effect upon my own mind merely from hearing his alarming fear of these things and the danger of appendicitis as the result made me very careful for some time, and had I been a nervous subject I can quite believe I might have frightened myself into it—fear always reduces the power of resistance.

I am quite aware that anybody doubting the value of vaccines and vaccination for smallpox, to which you refer in this article, is at once written down as an ignorant crank. I have, however, been studying the Registrar-General's figures and other facts relating to smallpox, and I am bound to say, so far, I cannot find anything whatever in favour of the practice. I wish you could help me to find some proof. I have read everything I can find and can so far only imagine it to be a source by which disease is spread. I am not dogmatising on the subject, but enquiring.

I read your Journal regularly with interest.

Robert Blackie.

National Liberal Club, S.W. 1.

